# Ecological patterns in the skin microbiota of frogs from tropical Australia

**DOI:** 10.1002/ece3.4518

**Published:** 2018-09-27

**Authors:** Keith Christian, Chava Weitzman, Alea Rose, Mirjam Kaestli, Karen Gibb

**Affiliations:** ^1^ Research Institute for the Environment and Livelihoods Charles Darwin University Darwin Northern Territory Australia; ^2^ Department of Biology University of Nevada Reno Nevada

**Keywords:** amphibians, arboreal, core microbes, frog skin, skin microbiota, terrestrial

## Abstract

The microbiota of frog skin can play an important role in protecting against diseases and parasites. The frog skin microbial community represents a complex mix of microbes that are promoted by the chemical environment of the frog skin and influenced by the animal's immediate past environment. The microbial communities of six species of frogs sampled from the campus of Charles Darwin University (CDU) were more similar within species than between species. The microbiota of the introduced cane toad (*Rhinella marina*) was most dissimilar among the species. Pairwise comparisons showed that the microbial communities of each species were different, except for the terrestrial *Litoria nasuta* and the arboreal *L. rothii*. The microbial communities of the six species were not related to ecological habit (arboreal or terrestrial), and neither was the alpha diversity of the microbes. The core microbes (defined as being on ≥90% of individuals of a species or group) were significantly different among all species, although 89 microbial operational taxonomic units (OTUs) were core microbes for all six species at CDU. Two species, *Rhinella marina* and *Litoria rothii*, were sampled at additional sites approximately 10 and 30 km from CDU. The microbial communities and the core OTU composition were different among the sites, but there were nevertheless 194 (*R. marina*) and 181 (*L*. *rothii*) core OTUs present at all three sites. Thus, the core microbiota varied with respect to geographic range and sample size.

## 1 INTRODUCTION

The moist skin of amphibians supports a diverse array of bacteria that play critical ecological roles, including defense against pathogens. Recent studies have shown that microbes on frog skin not only confer protection against skin diseases and other benefits (Walke et al., 2014; Knutie, Wilkinson, Kohl, & Rohr, 2017; McFall‐Ngai et al., 2013), but that the microbiota is, to some extent, characteristic for a species even among coexisting species (McKenzie, Bowers, Fierer, Knight, & Lauber, 2012; Kueneman et al., 2013; Walke et al., 2014). Like other recently explored microbiota, communities on frog skin are the result of complex processes involving both environmental influences and host‐specific characteristics (Adair & Douglas, 2017), and thus, environment (location) can also be a significant factor in determining bacterial community composition (Kueneman et al., 2013).

Environmental factors that can influence or disrupt the microbial community on amphibian skin include the season (Woodhams et al., 2014; Longo, Savage, Hewson, & Zamudio, 2015; Longo & Zamudio, 2016), temperature (Daskin, Bell, Schwarzkopf, & Alford, 2014; Wu, Cramp, & Franklin, 2017), water pH (Krynak, Burke, & Benard, 2015), contaminants (Costa, Lopes, Proença, Ribeiro, & Morais, 2016), and captivity (Loudon et al., 2014; Becker, Richards‐Zawacki, Gratwicke, & Belden, 2014; Sabino‐Pinto et al., 2016; Kueneman et al., 2016). Biologic factors include transitions in life stages (Rollins‐Smith, Ramsey, Pask, Reinert, & Woodhams, 2011; Kueneman et al., 2013; Bresciano et al., 2015; Sanchez et al., 2017), diet (Antwis et al., 2014), sloughing the skin (Meyer, Cramp, Bernal, & Franklin, 2012; Wu et al., 2017), disease (Jani & Briggs, 2014), and microbial interactions (Loudon et al., 2016; Bates et al., 2018).

Despite the perturbations resulting from these environmental and biologic factors, species‐specific communities persist and are consistent in different locations (McKenzie et al., 2012; Kueneman et al. 2013; Walke et al., 2014; Belden et al., 2015) and prolonged captivity (Becker et al., 2014). This apparent homeostasis is consistent with the notion that several characteristics of the amphibian skin (epidermal structures, skin peptides, and other mucosal components) select and enhance specific bacteria over others (Rollins‐Smith & Woodhams, 2012). This dynamic process between a disruptive environment and the homeostatic properties of the skin can explain the seemingly contradictory reports of both variability and consistency of the microbiota of amphibian skins (Kueneman et al., 2013; Adair & Douglas, 2017).

Much of the research pertaining to frog skin microbiota has focused on the complexities of the epidemic of chytrid fungus (Rollins‐Smith et al., 2011; Jani & Briggs, 2014; Holden et al., 2015; Berger et al., 2016; Bates et al., 2018) and other frog skin diseases (Federici et al., 2015; Knutie et al., 2017), including suggestions of using probiotics on frog populations (Harris et al., 2009; Loudon et al., 2014; Küng et al., 2014) even at a landscape scale (Muletz, Myers, Domangue, Herrick, & Harris, 2012). These efforts have yielded inconclusive results, including problems associated with the resilience of the skin microbiota inhibiting the uptake of probiotics (Küng et al., 2014). Recent reviews have cautioned against this highly targeted approach, calling for studies to enhance the understanding of the ecological and evolutionary context in which frog skin microbiota operates (Kueneman et al., 2013; Küng et al., 2014; Kueneman et al., 2015; Woodhams, Bletz, Kueneman, & McKenzie, 2016).

Detailed exploration of host–microbiota associations has resulted in the concept of a “core” community, based on near ubiquity among individuals of a host species. The core microbiota has been variously defined, but is typically defined as being present on >90% of hosts (Loudon et al., 2014; Apprill et al., 2014).

The aim of this study was to enhance our understanding of the ecological context of the skin microbiota by sampling from a previously well‐studied group of frogs (Tracy & Christian, 2005; Young, Christian, Donnellan, Tracy, & Parry, 2005; Tracy, Christian, Betts, & Tracy, 2008; Tracy, Tixier, Le Nöene, & Christian, 2014) from the wet–dry tropics of Australia. We studied six frog species from one site, three of which are arboreal species and three of which are terrestrial. Previous studies of this community of frogs have shown that ecological habit (arboreal versus terrestrial) is associated with some physiological traits in this seasonal tropical environment (see references cited above). To explore the effect of host species, ecological habit, and geographic location on skin microbial community patterns, we further sampled two of the six species from two additional sites at distances of approximately 10 km and 30 km from the main study site.

## 2 MATERIALS AND METHODS

### 2.1 Species, study sites, and sampling scheme

**Figure 1:**
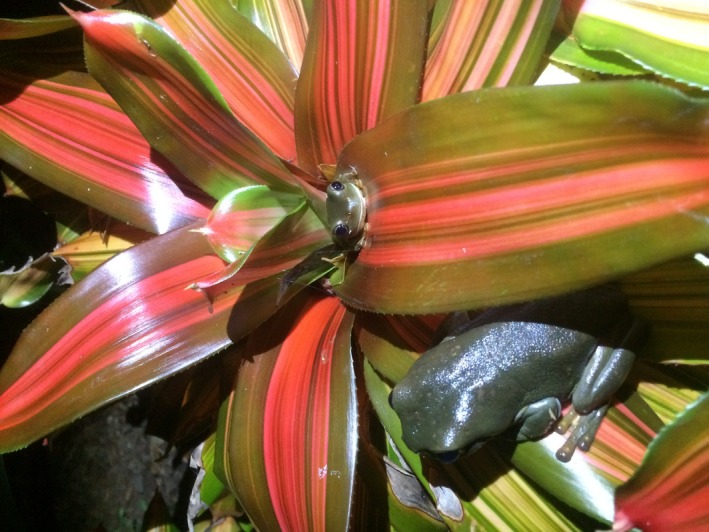
Two green tree frogs, *Litoria caerulea*, on the campus of Charles Darwin University, Darwin, Northern Territory, Australia

Approval to sample frogs was granted by the Charles Darwin University Animal Ethics Committee (project A14012). Three terrestrial frogs (*Rhinella marina*,* Litoria nasuta*, and *Limnodynastes convexiusculus*) and three arboreal frogs (*Litoria caerulea* (Figure 1)*, Litoria rubella*, and *Litoria rothii*) species were sampled from on or near the campus of Charles Darwin University (CDU), in Darwin, Northern Territory, Australia. Although some frogs were collected near buildings on campus, others were sampled from a more natural area consisting of native vegetation. Two species, the cane toad, *Rhinella marina*, and Roth's tree frog, *Litoria rothii*, were also sampled from Mickett Creek and Howard River, approximately 10 and 30 km, respectively, from CDU. These rural areas primarily consist of native vegetation, with widely dispersed buildings. The cane toad was introduced to Australia approximately 80 years ago (Easteal, 1981), but the other species are native to the area. Twenty individual frogs were sampled from each species and location (total of 200 samples). New gloves were used for each sample.

After capture by hand, each frog was rinsed twice with 100 mL 0.45 μm filtered high‐purity water (Culp, Falkinham, & Belden, 2007; Lauer et al., 2007) before being swabbed with a sterile synthetic swab (MicroRheologics FLOQSwab). Each frog was stroked 30 times to produce a sample, using 10 strokes around body (avoiding cloaca, 4 dorsal, 1 each side, 4 ventral) and 5 strokes on each limb (front and back of the foot, front and back of the leg, axial region). Samples were kept on ice while in the field and then frozen at −20°C until the DNA was extracted.

### 2.2 DNA extraction

DNA was extracted from the samples using the Qiagen DNeasy Blood and Tissue Kit (Qiagen, Valencia, CA), following the manufacturer's protocol for tissue extraction.

### 2.3 16S rDNA target sequencing

Two hundred nanograms of DNA was sent to the sequencing provider Molecular Research DNA (http://www.mrdnalab.com, Shallowater, TX, USA) for amplification using the Caporaso et al. (2011) primers, F515 (GTGCCAGCMGCCGCGGTAA) and R806 (TAATCTWTGGGVHCATCAGG) targeting the V4 variable region of the 16s rRNA gene. The forward primers contained sample‐specific eight‐nucleotide barcodes. A 30‐cycle PCR using the HotStarTaq Plus Master Mix Kit (Qiagen) was run with the following conditions: 94°C for 3 min, 28 cycles of 94°C for 30 s, 53°C for 40 s, 72°C for 1 min, and final elongation step at 72°C for 5 min. Amplicon products from different samples were mixed in equal concentrations and purified using Ampure XP beads (Agencourt Bioscience Corporation: Beverly, MA). Pooled and purified PCR products were used for DNA library preparation according to the Illumina TruSeq DNA library preparation protocol. Samples were sequenced utilizing a MiSeq instrument, following manufacturer's guidelines.

### 2.4 Processing of sequencing data

Sequence data were processed using a proprietary analysis pipeline (http://www.mrdnalab.com, Molecular Research DNA, Shallowater, TX). Sequences were depleted of barcodes, and primers and short sequences <200 bp were removed as well as sequences with ambiguous base calls and homopolymer runs exceeding 6nt. Chimeras were also removed. Sequences were clustered at 3% divergence (97% similarity) to define operational taxonomic units (OTUs), and OTUs with singleton sequences were removed (Dowd et al., 2008; Edgar, 2010; Swanson et al., 2011; Capone, Dowd, Stamatas, & Nikolovski, 2011). OTUs were taxonomically classified using BLASTn against a curated Greengenes database (DeSantis et al., 2006).

Furthermore, OTUs were excluded which were not classified as bacteria, occurred in less than 1% of samples (i.e., in less than three samples), or contained fewer sequences than 0.01% of the total sequence abundance. All sequences were subsampled to the lowest common sequence number (2,008 sequences) per sample.

### 2.5 Data analysis

operational taxonomic unit data were analyzed in Primer‐7 (Clarke & Gorley, 2001; Primer‐E, Plymouth, UK) and in R (version 3.2.2.) using the packages phyloseq in Bioconductor (Callahan, Sankaran, Fukuyama, McMurdie, & Holmes, 2016), corrplot, vennerable, and labdsv.

The dataset was subset into two groups: Frogs sampled on or near the CDU campus and the cane toads and Roth's tree frogs from three sites (CDU, Mickett Creek, and Howard River). Bacterial orders that occurred at relative abundances of more than 1% in a frog species or site are shown in taxa plots using phyloseq. A weighted UniFrac distance matrix was created. The distance matrix was visualized using nonmetric multidimensional scaling (nMDS) and a triangle heat map. Alpha diversity and changes in the frog skin microbial communities were analyzed in Primer‐7 by permutational MANOVA (PERMANOVA; 9999 permutations) with fixed factors “frog species” and “habitat” for the CDU frog dataset and “frog species” and “site” for the toad and Roth's tree frog dataset. Pairwise tests for frog species, habitat, and site were also conducted if the main test was significant (*p *<* *0.05). Alpha diversity was examined with respect to ecological habit using a linear mixed‐effect model in Stata with ecological habit as a fixed effect and frog species as a random effect nested in ecological habit.

We identified the core microbiota for campus frogs, and toad and Roth's tree frogs sampled at the campus, Howard River, and Mickett Creek sites by selecting those OTUs that were present in at least 90% of samples within a group. Core OTUs are shown in Venn diagrams, and PERMANOVA and pairwise analyses were also conducted on these core OTUs to explore significant differences in the core microbiota between frog species and sites.

## 3 RESULTS

### 3.1 Microbial community composition

**Figure 2:**
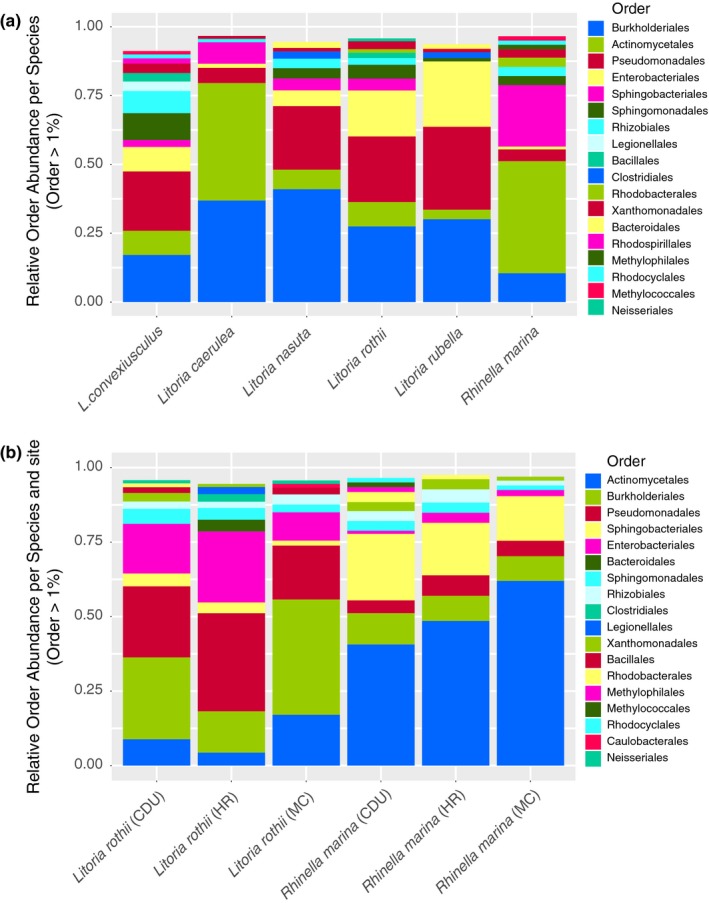
Major skin microbial orders which occurred at more than 1% in (a) six species of frogs sampled from the campus of Charles Darwin University (CDU) (*n* = 20 for each species) and (b) two species (*L. rothii and R. marina*) sampled from CDU, Mickett Creek (MC), and Howard River (HR), approximately 10 and 30 km from CDU, respectively (*n* = 20 per species per location)

After data processing, 651 bacterial OTUs were recorded from the six frog species sampled. The composition of frog skin bacteria at the level of order is shown in Figure 2a. The most dominant orders included Burkholderiales, Actinomycetales, Pseudomonadales, Enterobacteriales, and Sphingomonadales. The same five orders were dominant in the samples from the two species sampled across three sites (Figure 2b). Visual inspection of Figure 2a reveals that *L. caerulea* and *R. marina* had considerably more (proportionally) Actinomycetales but fewer Pseudomonadales and Enterobacteriales than the other frogs.

The alpha diversity, as represented by the Shannon index, is given in Table 1 for both the total complement of OTUs and the core OTUs for the six species on the CDU campus. The diversity of OTUs was significantly higher in *Limnodynastes convexiusculus* (4.97) than in the other frog species (*F*_5,119_ = 31.3, *p *<* *0.0001), and this was also true for the diversity of core OTUs. The lowest diversity (3.10) was found in *Litoria caerulea*, and this species also had the lowest diversity of core OTUs. Once the effect of species was taken into account, there was no significant effect of ecological habit on alpha diversity (*p *=* *0.24).

**Table 1:** (A) The total number of OTUs and core OTUs measured for each of six species sampled from the campus of Charles Darwin University (CDU) and for two species at two additional sites. The %Core is defined as the number of core OTUs expressed as a percentage of the total number of OTUs. The Shannon diversity index is used to represent the total diversity and core diversity for the frogs at CDU. (B) The core is compared using data from three sites for two species. The combined core treats the data from three sites as one group. Thus, combined core OTUs were found in at least 90% of all individuals of a species as opposed to 90% of individuals from a given site

A											
Species	CDU campus					Mickett Creek			Howard River		
	Total OTUs	Total diversity	Core OTUs	Core diversity	%Core	Total OTUs	Core OTUs	%Core	Total OTUs	Core OTUs	%Core
*Rhinella marina*	635	3.48	281	3.45	44.2	637	256	40.2	635	278	43.8
*Litoria rothii*	651	4.02	270	3.99	41.5	651	256	39.3	651	268	41.2
*L. caerulea*	651	3.10	244	3.09	37.5						
*L. rubella*	644	3.49	236	3.45	36.6						
*L. nasuta*	651	3.96	343	3.93	52.7						
*Limnodynastes convexiusculus*	651	4.97	460	4.95	70.7						

B			
Species	Total OTUs	Combined core OTUs	% Combined core of total
*Rhinella marina*	645	194	30.0
*Litoria rothii*	651	181	27.8

### 3.2 Frog species comparisons

**Figure 3:**
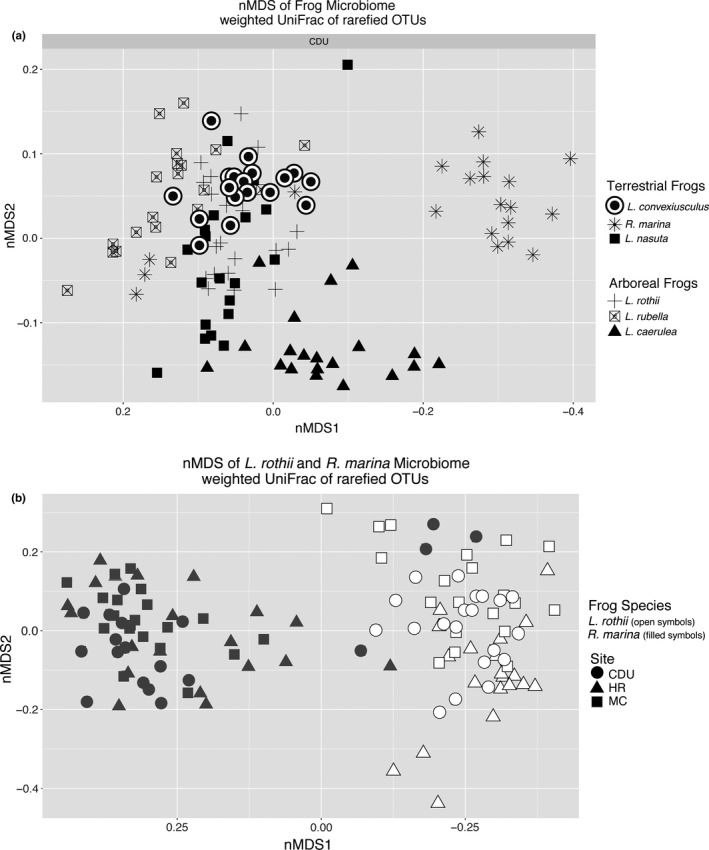
Relatedness of the skin microbiota as shown by a nonmetric multidimensional scaling (nMDS) of (a) six frog species sampled from the campus of Charles Darwin University (CDU) and (b) two species (*L. rothii and R. marina*) sampled from CDU, Mickett Creek (MC), and Howard River (HR). Each nMDS was based on the weighted UniFrac distance matrix of rarefied OTU data and had a stress value <0.14 (a) and <0.10 (b)

An nMDS showed clustering of the microbial composition by frog species (Figure 3a), and PERMANOVA confirmed the clustering of communities according to species (*F*_5,119_ = 36.1, *p *<* *0.001). The microbial communities of the frog species were significantly different from one another in pairwise comparisons (*p *<* *0.001), except not between the terrestrial *Litoria nasuta* and the arboreal *L. rothii*.

**Figure 4:**
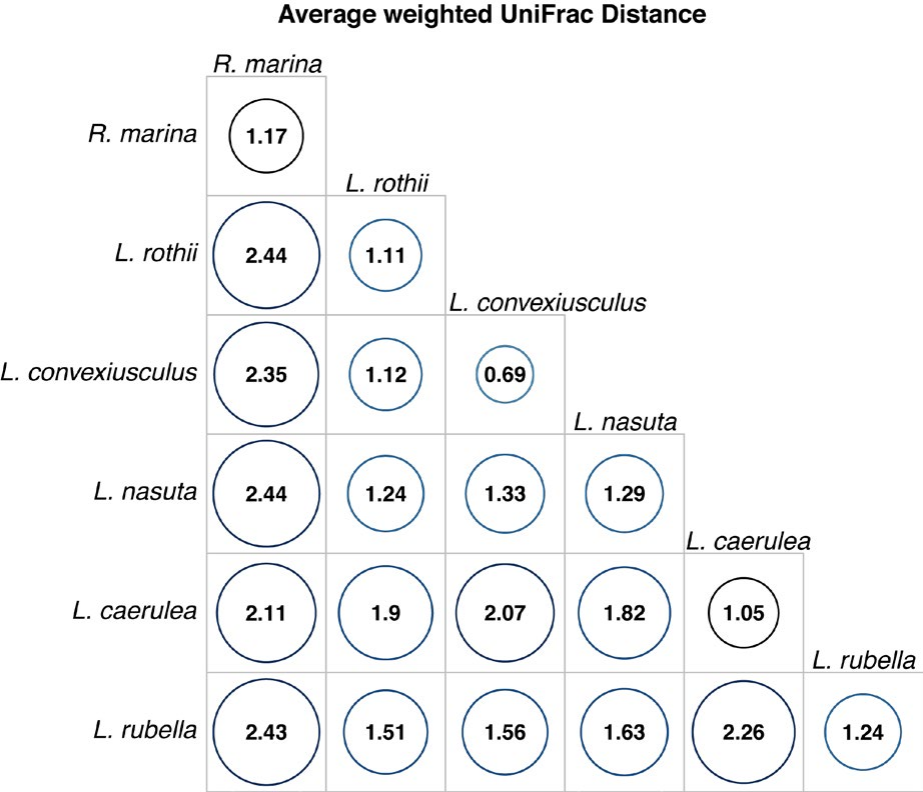
Heat map triangle showing the average weighted UniFrac distances (dissimilarities of the OTU data between species). The circles on the diagonal show the within‐species dissimilarities from the 20 individuals of each of the six frog species. Larger circles and numbers represent greater dissimilarities within and between species

A triangle heat map of average weighted UniFrac distances between frog species illustrates that the skin microbiota of the introduced cane toads was the most dissimilar when compared to the five native species, and communities were more similar within species than between species (Figure 4). Microbial communities of the three terrestrial species and the three arboreal species were significantly different between the two ecological habits (*F*_1,119_ = 12.5, *p *<* *0.0001).

The microbial communities of *Rhinella marina* and *Litoria rothii* (Figure 3b) were significantly different across the three sites over 30 km (*F*_2,119_ = 4.0, *p *<* *0.009). Pairwise comparisons showed that *Litoria rothii* communities were different at all three sites (*p *<* *0.003) and *Rhinella marina* communities were different between CDU and Mickett Creek and between Howard River and Mickett Creek (*p *<* *0.02), but not between CDU and Howard River (*p *=* *0.48).

### 3.3 Core OTUs

**Figure 5:**
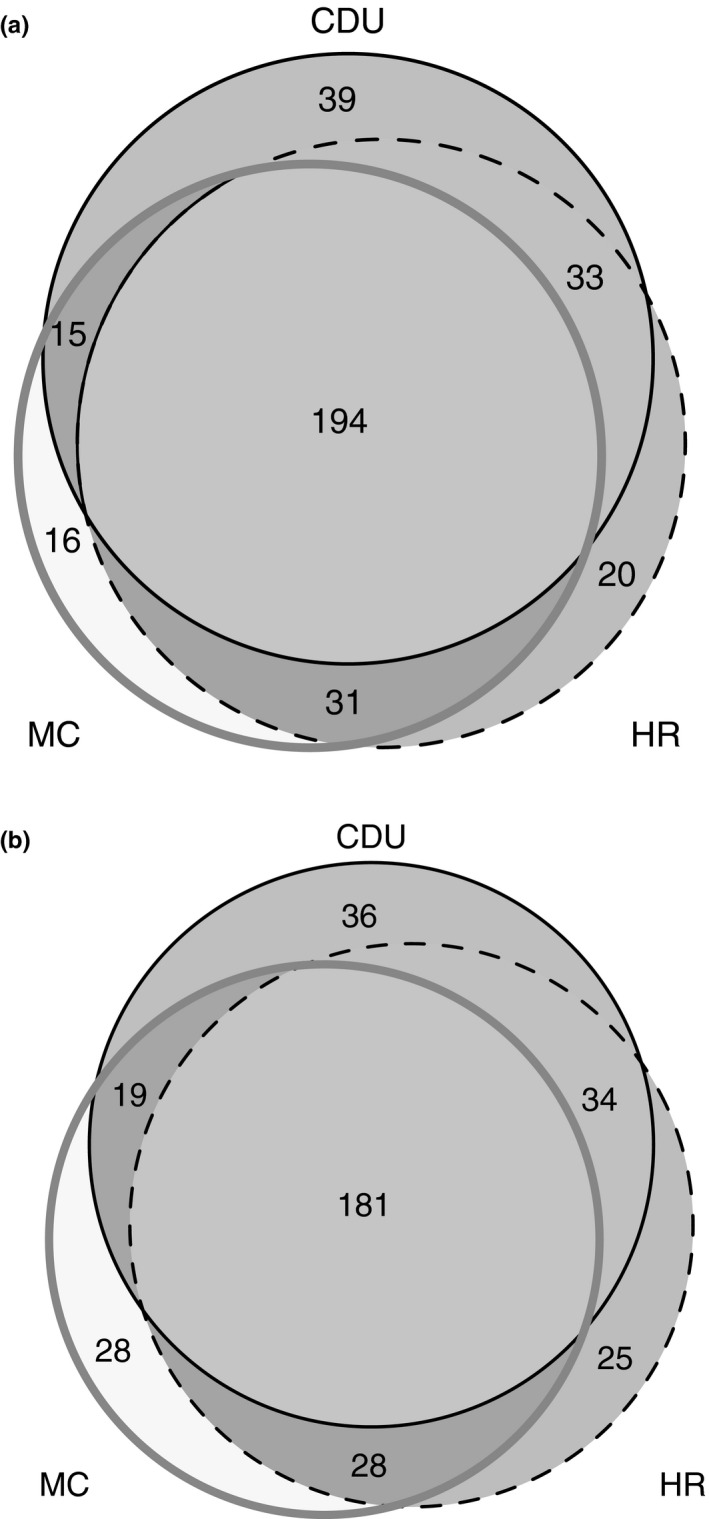
Venn diagrams showing the number of core OTUs across three sites (Charles Darwin University (CDU), Mickett Creek (MC), and Howard River (HR)) for (a) *R. marina* and (b) *L. rothii*

The number of core OTUs for each frog species is listed in Table 1, and the core OTUs are also expressed as a percentage of the total OTUs for each frog species. Overall, 604 OTUs were core OTUs for at least one frog species. Over 70% of the total OTUs in *Limnodynastes convexiusculus* were core OTUs, which was a distinctly higher percentage than in the other frogs. *Litoria rubella* had the lowest percentage as core OTUs (36.6%) among the six species. The composition of the core OTUs was significantly different between frog species at CDU (*F*_5,1452_ = 164.1, with *p *<* *0.001 for all pairwise comparisons), although 89 core OTUs were shared by all six species on the CDU campus. The core OTUs for *R. marina* and *L. rothii* were significantly different across three sites (*F*_2,509_ = 9.3, *p *<* *0.0007), but there were nevertheless 194 OTUs found on *R. marina* at all three sites (Figure 5a) and 181 OTUs found on *L. rothii* at all three sites (Figure 5b).

## 4 DISCUSSION

The six frog species studied had species‐specific microbial communities on their skin, despite those at CDU being from the same location. Strikingly, *L. caerulea* and *L. rubella,* many of which were collected from the same part of campus, had distinctly different skin microbial communities (Figure 3a). Species‐specific microbial skin communities have been found in previous studies *(McKenzie* et al.*,* 2012 *; Belden* et al.*,* 2015; Rebollar et al. 2016), but this example is noteworthy because of their geographic proximity, they shared ecological habit (arboreal), and they are congeneric. The microbial community on the introduced cane toad, *R. marina*, was distinctly different from the two native terrestrial species (*Litoria nasuta* and *Limnodynastes convexiusculus*) (Figure 3a), and it was the most dissimilar in comparison with all the other species (Figure 4).

The five dominant orders represented on the skin have also been reported in other amphibian studies, particularly Burkholderiales, which include many common soil bacteria, and Actinomycetales, which are not only common in soil, but which produce bioactive metabolites with antibiotic activity (Bates et al., 2018). Kueneman et al. (2015) and Bataille et al. (2016) reported the numerical dominance of both Actinomycetales and Sphingomonadales on the skin of toads and their possible antifungal role. The Pseudomonadales, particularly those of the genus *Pseudomonas*, contain several species known to provide protection against pathogenic bacteria and fungi (Chang, Huang, Lin, Huang, & Liao, 2016; Federici et al., 2015). The Enterobacteriales were dominant in this study, and while this order has been reported from the guts of frogs (Chang et al., 2016), we found only two reports from amphibian (toad) skin studies (Hughey et al., 2017; Bataille et al. 2016). Enterobacteria can be found almost everywhere (soil, water, wastewater, animal guts), so frogs could come into contact with them from drains and water bodies (Neave et al., 2014) or via invertebrates they have consumed (Chang et al., 2016).

Taken as a group, the communities of arboreal frogs were significantly different from terrestrial frogs, as are a range of physiological characteristics (Young et al., 2005; Tracy et al., 2014). Nevertheless, the microbial community of the terrestrial frog *L. nasuta* was not different from that of the arboreal *L. rothii*, suggesting that the significant difference between the two ecological habits was, in part, due to the general pattern of frog species‐specific microbial communities. The similarity between *L. nasuta* and *L. rothii* cannot be explained by phylogenetic relationships because the arboreal species *L. rothii* and *L. rubella* are closely related, but *L. nasuta* is more distantly related and groups with other terrestrial species in the genus (Young et al., 2005).

The number of core OTUs for the six frog species on the CDU campus ranged from 256 to 281 (Table 1), and these represented 36.6% to 70.7% of the total microbiota for the frog species, with the core OTUs of *Limnodynastes convexiusculus* being the highest percentage of the total. For the two species sampled across three sites, the core was variable among the sites, yet there were nevertheless OTUs that were present on all individuals over the 30‐km transect (Figure 5). If the data from three sites are grouped within a species, the number of OTUs that meet the definition of “core” is substantially smaller than the number of core OTUs from a single site. This raises questions about the definition and determination of the core microbiota. One could argue that the best measurement of the core microbes is represented by the central portion of the Venn diagrams in Figure 5, which is not only based on a larger sample (*n* = 60), but the larger geographic range of the sample also likely provides a more comprehensive characterization of the microbiota. However, even if one accepts this argument over a 30‐km transect, the definition and determination of the core microbiota become problematic over greater distances because both the environment and the frogs themselves could vary substantially at a larger (i.e., continental) scale. Thus, the optimal microbiota at one site may not be the same at another site with frogs and microbes adapted to each local environment.

The core microbiota is defined on the basis of prevalence (≥90% of individuals), but the unstated assumption is that if it is found in most individuals, then a core microbe is likely to have a role in the microbiota in producing important metabolites that are either important to the frog (i.e., antifungal properties or activation of the immune system (McFall‐Ngai et al. 3013)) or in structuring the microbiota. However, near ubiquity in the skin microbiota (i.e., a core microbe) could reflect either functional importance or simply prevalence in the environment. However, the fact that different frog species from the same location have different cores provides indirect support for the notion that the core microbiota is not simply reflecting the ubiquity of microbes in the environment. Analyses that indicate microbial function in the microbiota (Berry & Widder, 2014) are more appropriate for determining the importance of an OTU (i.e., a “keystone”) in a microbiota. The relationship between core and keystone OTUs has received little attention. Figure 5 indicates that a core microbe (as determined at a site level) may not be functionally important (given that it is not part of the core at sites 10 or 30 km away), but rather, some environmental microbes may be abundant at some sites but not others. However, is the converse true? That is: Is it possible for a keystone microbe to not be a core microbe? Understanding the functional importance of microbes within the microbiota is crucial to advancing our understanding of these relationships. Although descriptively satisfying, the concept of a core (as currently defined) may not continue to be a useful construct as our understanding of the structure and function of the frog skin microbiota develops.

## CONFLICT OF INTEREST

None declared.

## AUTHOR CONTRIBUTIONS

K.C., K.G., and C.W. designed the project, K.C. and C.W. collected the samples in the field, C.W. extracted the DNA, M.K. and A.R. analyzed the data and produced the figures, and K.C., with assistance from all other authors, wrote and revised the manuscript.

## DATA ACCESSIBILITY

Sequences from this study have been deposited via Qiita to the European Nucleotide Archive (ENA), the permanent data repository of the European Bioinformatics Institute (EBI): STUDY ID: 11481, SAMPLE: 082614KG359F, EXPERIMENT: 16S rRNA sequenced skin microbial communities, and RUN: 121815KG515Fcomplete‐pr.fasta (121815KG515F). Available via Qiita StudyFilter: https://qiita.ucsd.edu/study/description/11481 .
